# Bruch einer Spinalnadel in situ im Rahmen einer elektiven Sectio caesarea

**DOI:** 10.1007/s00101-023-01358-1

**Published:** 2023-11-10

**Authors:** Josefin Grabert, Christian Klebach, Isabelle Osberghaus, Patrick Jakobs, Se-Chan Kim, Brigitte Strizek, Mark Coburn, Tobias Hilbert

**Affiliations:** 1 Klinik und Poliklinik für Anästhesiologie, und Operative Intensivmedizin, Universitätsklinikum Bonn, Venusberg-Campus 1, 53127 Bonn, Deutschland; 2 Zentrum für Anästhesie, Perioperative Medizin und Schmerztherapie, Orthopädische Klinik Markgröningen, Markgröningen, Deutschland; 3 Zentrum für Geburtshilfe und Frauenheilkunde, Abteilung für Geburtshilfe und Pränatale Medizin, Universitätsklinikum Bonn, Bonn, Deutschland

**Keywords:** Sectio caesarea, Spinalanästhesie, Nadelbruch, Rückenmarksnahe Regionalanästhesie, Geburtshilfe, Cesarean section, Spinal anesthesia, Broken needle, Neuraxial regional anesthesia, Obstetrics

## Falldarstellung

### Anamnese

Bei einer 36-jährigen Schwangeren (Gravida 1, Para 0) ist auf Wunsch der Patientin eine primäre Sectio caesarea geplant. Relevante Begleiterkrankungen umfassen eine Adipositas per magna (Body Mass Index (BMI) 48 kg/m^2^) sowie eine Faktor-V-Leiden-Mutation. Die präoperativ bestimmten Laborwerte zeigen keine pathologischen Befunde (Partielle Thromboplastinzeit (PTT) 25 s; Quick-Wert 117 %; Thrombozyten 319 G/l; Hämoglobin 12,8 g/dl).

Nach Installation des Routinemonitorings (bestehend aus 5‑Pol-EKG, Pulsoxymetrie und nichtinvasiver Blutdruckmessung) sowie Anlage eines peripheren Venenverweilkatheters wird die Patientin auf dem OP-Tisch in die sitzende Position gebracht.

### Klinischer Befund

Aufgrund der Adipositas der Patientin ist das regelrechte Ertasten des Beckenkammes erschwert, jedoch kann ein Punktionsort in Höhe LWK 3/4 ohne Zuhilfenahme der Sonographie bestimmt werden. Nach sterilem Abdecken, Vorbereitung des Materials und der Durchführung einer Lokalanästhesie mit 30 mg Mepivacain s.c. wird durch die Assistenzärztin im Beisein eines Facharztes ein erster, jedoch erfolgloser Punktionsversuch mit einer 25-G-Pencil-Point-Kanüle (Länge 88 mm; Fa. B. Braun, Melsungen, Deutschland) über eine 20-G-Führungskanüle (50 mm) unternommen. Dabei wird die Spinalkanüle ohne Knochenkontakt maximal tief eingeführt; Liquorrückfluss bleibt jedoch aus. Die Spinalnadel wird entfernt, die Führungskanüle belassen. In der Annahme einer nichtausreichenden Länge der Spinalnadel wird über dieselbe Führungskanüle nun eine längere Spinalkanüle (103 mm, 25 G; Fa. B. Braun) vorgeführt. Auch hier kommt es bei fehlendem Knochenkontakt zu einer Punctio sicca. Daher wird nun die Führungskanüle mitsamt der Spinalnadel gezogen. Dabei zeigt sich, dass Letztere exakt am distalen Ende der Führungskanüle abgebrochen ist, sodass der Großteil der Spinalnadel (ca. distale 55 mm) *in situ* verblieben sein muss.

### Verlauf

Die Patientin präsentiert sich allzeit ohne neurologische Auffälligkeiten. Kribbelparästhesien oder auch Schmerzen treten nicht auf. Der Versuch, das *in situ* verbliebene Nadelstück vorsichtig in der Tiefe zu ertasten, bleibt erfolglos.

Umgehend wird der Oberarzt hinzugezogen und die Patientin über das Geschehen informiert. Im interdisziplinären Konsens zwischen Anästhesie, Geburtshilfe und Neurochirurgie wird bei derzeit vollkommen asymptomatischer Patientin entschieden, zunächst die Sectio und im Anschluss eine bildgebende Diagnostik durchzuführen. Man entscheidet sich unter Zustimmung der Patientin für einen erneuten spinalen Punktionsversuch auf anderer Höhe.

Vor dem erneuten Punktionsversuch wird zunächst sonographisch das *in situ* verbliebene Nadelstück dargestellt. Das abgebrochene Fragment kann lateral der Wirbelkörper auf Höhe L3/4 dargestellt werden. Die erneute Punktion wird durch den Oberarzt mit einer kaliberstärkeren Pencil-Point-Nadel (22 G, 120 mm; Fa. B. Braun) durchgeführt. Für diese Nadel wird aufgrund des größeren Durchmessers (0,7 mm [vs. 0,53 mm bei 25 G-Spinalnadel]) keine Führungskanüle verwendet. Nach mehreren Punktionsversuchen sowie Lagekorrekturen der Nadel kann schließlich in Höhe LWK 2/3 ein Rückfluss von unauffälligem Liquor cerebrospinalis erzielt und unter 2‑maliger positiver Barbotage die Spinalanästhesie mit 8,5 mg Bupivacain hyperbar sowie 5 µg Sufentanil erfolgreich etabliert werden. Ein fühlbarer Kontakt zu dem abgebrochenen Nadelfragment bestand dabei zu keinem Zeitpunkt. Die Anästhesie steigt in der Folge regelrecht auf. Der weitere Verlauf der Schnittentbindung ist komplikationslos. Ungewöhnliche Symptome seitens der Patientin, die auf Probleme durch das Nadelfragment hinweisen könnten, treten ebenfalls nicht auf.

**Figure 1:**
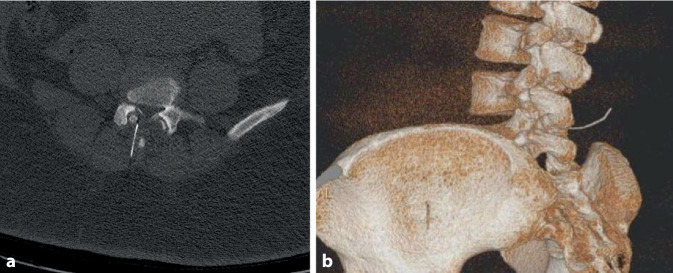

**Figure 2:**
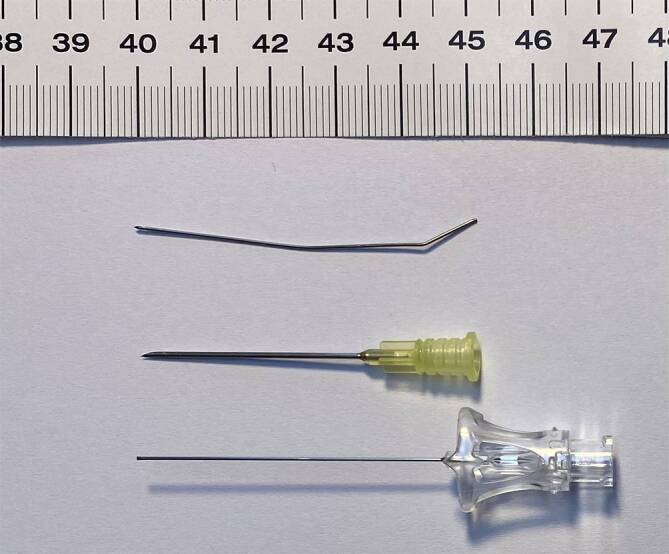

Im Anschluss an die Operation wird bei der Patientin zur genauen Lokalisation des abgebrochenen Nadelfragments ein Computertomogramm (CT) der Lendenwirbelsäule durchgeführt. Es zeigt sich ein ca. 4 cm langes Fragment, das mit der Spitze an den rechten Pedikel von LWK 5 reicht und ab dem anderen Ende etwa 6 cm Abstand zur Haut hat (Abb. 1). Noch am Tag der Sectio wird dieses bei durchgehend asymptomatischer Patientin im Rahmen eines neurochirurgischen Eingriffs entfernt. Diese Operation wird in Bauchlagerung in Intubationsnarkose durchgeführt. Unter wiederholter intraoperativer Röntgenkontrolle kann das Nadelfragment *in toto* geborgen werden (Abb. 2). Eine weiterführende neurochirurgische Überwachung wird nicht notwendig, die Patientin wird nach Entlassung aus dem Aufwachraum durch die Geburtshilfe gemäß örtlichem Sectio-Standard weiterbetreut und kann am 4. postoperativen Tag symptomlos nach Hause entlassen werden. Auch mehrere Wochen nach dem Ereignis zeigt die Patientin keine neurologischen Beschwerden.

## Diskussion

Unter Beachtung von Kontraindikationen sowie Anamnese und Labor ist die Spinalanästhesie zur geplanten Sectio caesarea das Verfahren der Wahl. Der Bruch von Spinalnadeln *in situ* ist ein seltenes, in der Literatur nur in Fallberichten beschriebenes Ereignis. Die Inzidenz wird auf 1:5000 bis 1:11.000 geschätzt [1]. Ursachen und begünstigende Faktoren für diese Komplikation sollen im Folgenden anhand der beschriebenen Fallberichte diskutiert und auf den vorgestellten Fall angewendet werden.

Die Literaturrecherche ergibt 2 Übersichtsarbeiten mit insgesamt 23 Fällen frakturierter Spinalnadeln sowie einen weiteren Fallbericht [1–3]. Dabei kam es neben den 14 Nadelbrüchen im Rahmen von Sectiones auch zu 2 Brüchen von Tuohy-Nadeln im Rahmen einer PDA-Anlage zur Geburt. Die weiteren Fälle verteilen sich auf rückenmarknahe Regionalanästhesien anderer Disziplinen. Eine Gemeinsamkeit ist ein geringes Nadelkaliber: In 15 aus 24 Fällen misst das Nadelkaliber 25 G oder kleiner. Materialstudien zu axialen Kräften an Spinalnadeln zeigen, dass Nadeln kleineren Kalibers und ohne Mandrin instabiler sind und vermehrt deformieren [4]. Unterstützt wird dies von einer Untersuchung, die das Abweichungsverhalten verschiedener Spinalnadeln in ballistischem Gel untersucht hat. Nadeln kleineren Kalibers weichen durchschnittlich mehr von der Trajektorie ab [5]. Dies kann als vermehrte Nadeldeformierung in Abhängigkeit des Kalibers interpretiert werden. Dass das Kaliber nicht der alleinige Faktor ist, wird jedoch zum einen daran deutlich, dass auch Nadelbrüche bei Anlage von Periduralkathetern beschrieben sind, und im beschriebenen ballistischen Modell traumatische Nadeln bedeutend mehr abweichen [1, 5].

Im Alltag wird zur Nadelwahl mit niedrigem Kaliber argumentiert, dass bei stärkerem Kaliber die Inzidenz des postpunktionellen Kopfschmerzes ansteigt. Dies gilt für traumatische Kanülen mit scharfem Schliff [6]. Bei atraumatischen Kanülen ist die Inzidenz des postpunktionellen Kopfschmerzes in Abhängigkeit des Kalibers nicht mehr nachweisbar [6]. Aus diesen Überlegungen könnte man ableiten, dass eine atraumatische Nadel mit größerem Kaliber das kleinste Risiko für Nadelbrüche hat, ohne ein relevant erhöhtes Risiko für postpunktionellen Kopfschmerz zu erzeugen. Systematische Studien dazu existieren aufgrund der geringen Inzidenz von Nadelbrüchen nicht. Im Hinblick auf den Nadelschliff wird in den amerikanischen Leitlinien jedoch die atraumatische Kanüle empfohlen.

Eindeutiger sind die Untersuchungen zum korrekten Umgang mit den Nadeln. Die kleinkalibrigen Nadeln sind durch einen Mandrin stabilisiert. Dieser soll solange in der Hohlnadel bleiben, wie diese vorgeschoben wird, und erst zurückgezogen werden, wenn die Dura perforiert ist. Die Perforation kann an einem feinen Widerstandsverlust erkannt werden. Ein vorheriges Herausnehmen, um die Durapunktion unmittelbar am Liquorrückfluss zu erkennen, ist nicht probat, da die Nadel deutlich an Stabilität verliert [7]. Als zweiter Nachteil kann das Lumen durch Gewebestücke okkludiert werden.

Gleichfalls bedeutend ist der Umgang mit dem Introducer. Bei einliegendem Introducer ist eine Richtungsänderung der Spinalnadel nicht möglich und soll nicht durchgeführt werden. Durch die vorgegebene Richtung des Introducers werden vorwiegend axiale Kräfte auf die Nadel ausgeübt, die zu Verformung und in der Folge Brüchen führen können. Zur Änderung des Punktionswinkels muss der Introducer neu positioniert werden. Ausschlaggebend ist dann, Introducer und Nadel in einem zurückzuziehen. Sollte die Nadel über den liegenden Introducer zurückgezogen werden, besteht ein Risiko für Nadelschäden durch Abscheren am Introducer, die eine Instabilität bedingen können. Dies geht als Gemeinsamkeit aus der Fallsammlung von Rieg et al. hervor. Wie in Abb. 2 aus dem vorgestellten Fall zu sehen ist, befindet sich die Bruchstelle exakt am Ende des Introducers, wie es bei einem Abschertrauma beim Zurückziehen der Nadel zu erwarten wäre. Obwohl die Nadel des ersten Punktionsversuches entgegen der empfohlenen Vorgehensweise über den liegenden Introducer zurückgezogen wurde, brach dann tatsächlich eine andere, neue Nadel, die lege artis mit dem Introducer zurückgezogen wurde. Als Ursache ist vorstellbar, dass während der Punktion der Introducer bei liegender Spinalkanüle umpositioniert oder vorgeschoben wurde oder der bereits genutzte Introducer am Schliff verändert war und die neue Spinalkanüle beschädigt hat. Dies ist jedoch spekulativ und retrospektiv nicht mehr verifizierbar.

Elektronenmikroskopische Studien konnten zeigen, dass Mikrotraumen an Nadelspitze und -schaft auch dann entstehen können, wenn es keinerlei Knochenkontakt während der Punktion gab [8, 9]. Daher sollte bei einer Mehrfachpunktion auch ohne sichtbaren Schaden an der Nadel ein Wechsel auf eine frische Kanüle frühzeitig in Betracht gezogen werden. Mehrfachpunktionen treten häufiger bei unerfahrenem Personal auf, sodass auch der Ausbildungsgrad einen Einfluss haben kann [10].

Bei vermuteter schwieriger Punktion kann die vorherige sonographische Darstellung des Punktionsfensters in Betracht gezogen werden. Damit lassen sich bei entsprechender Vertrautheit mit der Methodik sowohl der Punktionswinkel als auch die Tiefe der Dura ermitteln. Ein Prädiktor einer schwierigen Punktion ist die Unmöglichkeit einer guten Positionierung der Patientin mit Rundrücken. Liegen vertebrale Deformitäten vor oder sind die anatomischen Landmarken nicht zu palpieren, ist ebenfalls von einer schwierigen Punktion auszugehen [10, 11]. Bezüglich des BMI ist die Datenlage widersprüchlich. Der BMI allein scheint nicht prädiktiv für eine schwierige Punktion zu sein, jedoch dann, wenn Positionierung und Palpation der Landmarken beeinflusst werden [11, 12]. Im hier beschriebenen Fall bestand bei der Patientin eine Adipositas permagna, bei problemloser Positionierung und palpablen Landmarken wurde initial von einer Sonographie abgesehen.

Kommt es trotz vorbeschriebenem Vorgehen zu einer derartigen Komplikation, sind im Umgang damit mehrere Optionen denkbar. Im hier vorgestellten Fall hat man sich trotz Nadelbruch zu Durchführung der Sectio und einer erneuten Spinalanästhesie entschieden. Dies ist zu betonen, da die Patientin nicht nur Prädiktoren einer schwierigen Punktion aufwies, sondern es bereits zu einer komplikativen Punktion gekommen war. In Anbetracht des erhöhten Risikos für eine Aspiration und erschwerte Atemwegssicherung ist, auch in Absprache mit der Patientin, ein weiterer spinaler Punktionsversuch durchgeführt worden. Dazu wurde sich für eine kaliberstärkere Nadel mit scharfem Schliff entschieden. Nach sonographischer Lokalisation des Fragments ließ sich dabei ein Kontakt mit diesem vermeiden, die Punktionstiefe abschätzen und eine adäquate Anästhesie zur Schnittentbindung erzielen.

Im vorgestellten Fall wurde das Nadelfragment zweizeitig, aber noch am selben Tag operativ geborgen. Die Patientin beklagte zu keinem Zeitpunkt Beschwerden. Auch bei fehlenden Symptomen sollte das Fragment geborgen werden, da durch eine Fragmentmigration das Risiko für sekundäre neurologische Defizite oder Schmerzen besteht. Die Bergung kann bei ausreichender Wirkung der Spinalanästhesie und in Abhängigkeit der OP-Ausstattung (Durchleuchtung) auch in gleicher Sitzung erfolgen [1, 13, 14].

## Fazit für die Praxis

- Nadelbrüche bei Spinalanästhesien sind ernste, aber glücklicherweise seltene Komplikationen.
- Aus bisherigen Berichten kann noch nicht eindeutig geschlossen werden, welchen Einfluss Kaliber und Schliff der Nadel haben. Ausschlaggebend ist jedoch der korrekte Umgang mit Nadel, Mandrin und Introducer.
- Eine Umpositionierung von einliegender Kanüle-Introducer-Kombination soll nicht erfolgen, ebenso das alleinige Zurückziehen der Spinalkanüle über einen liegenden Introducer.
- Bei Vorliegen von prädisponierenden Faktoren für eine schwierige Punktion (erhöhter BMI, besonders in Kombination mit erschwerter Positionierung und Palpation der Landmarken) kann die Sonographie hilfreich sein, erfordert jedoch entsprechende Übung.
